# Prevalence of COPD among population above 30 years in India: A systematic review and meta-analysis

**DOI:** 10.7189/jogh.11.04038

**Published:** 2021-08-21

**Authors:** Ashwani Verma, Nachiket Gudi, Uday N Yadav, Manas Pratim Roy, Amreen Mahmood, Ravishankar Nagaraja, Pradeepa Nayak

**Affiliations:** 1 Evidence Synthesis Specialist, Campbell South Asia, New Delhi, India; 2 DIT University, Dehradun, Uttarakhand, India; 3 Department of Health Policy, Prasanna School of Public Health, Manipal Academy of Higher Education, India; 4 Centre for Primary Health Care and Equity, University of New South Wales, Sydney, Australia; 5 Center for Research, Policy, and Implementation, Biratnagar, Nepal; 6 Department of Public Health, Torrens University, Sydney, Australia; 7 Deputy Assistant Director General, Directorate General of Health Services, Nirman Bhawan, New Delhi, India; 8 Department of Physiotherapy, Kasturba Medical College, Mangalore, Manipal Academy of Higher Education, Karnataka, India; 9 Department of Biostatistics, Vallabhbhai Patel Chest Institute, University of Delhi, Delhi, India; 10 Department of Physiotherapy, Manipal College of Health Professions, Manipal Academy of Higher Education, Manipal, Karnataka, India

## Abstract

**Background:**

By 2030, Sustainable Development Goal 3.4 aims to reduce the premature mortality caused by non-communicable diseases through prevention and treatment. Chronic obstructive pulmonary disease is the second leading cause of mortality and disability-adjusted life years in India. This review was conducted to estimate the prevalence of COPD using systematic review and meta-analysis technique.

**Method:**

Search was conducted using six databases for studies on COPD among population above 30 years in India between years 2000 to 2020. Cross-sectional and cohort studies reporting prevalence of COPD and associated risk factors were included in the present review. Screening and data extraction was done by two authors independently. Studies were appraised for quality using the modified New Castle Ottawa scale and reporting quality was assessed using STROBE guidelines.

**Result:**

Our search returned 8973 records, from which 23 records fulfilled the eligibility criteria. Overall, the prevalence of COPD among population aged 30 years and above in India was 7%. Risk factors like active and passive smoking, biomass fuel exposure, environmental tobacco smoke, occupational exposure to dust, indoor and outdoor pollution, and increasing age were reported to have a significant association with COPD among Indian population.

**Conclusion:**

Our findings suggest the need for a multicentric national-level research study to understand COPD burden and its contributing risk factors. The findings also suggest the need for COPD sensitive health literacy program focused on early screening and primary prevention of risk factors for COPD, which may help early initiation of self-management practices, that are crucial for better quality of life.

Chronic Obstructive Pulmonary Disease (COPD) is a “common preventable, and treatable disease that is characterized by persistent respiratory symptoms and airflow limitation due to abnormalities in the airway and (or) alveolar abnormalities usually caused by significant exposure to noxious particles or gases” [1]. According to the World Health Organization (WHO), COPD is among the leading causes of deaths globally [2]. The WHO Global Alliance against Chronic Respiratory Diseases is committed towards the common goal to reduce the global burden of respiratory diseases [3].

According to the WHO’s estimates, nearly 65 million people have moderate to severe COPD that accounts for 5% of deaths (41.9 deaths per 100 000 individuals) globally and COPD remained the most prevalent disease-specific chronic respiratory disease (CRD) in 2017 [4,5]. COPD also imposes a significant burden owing to high health care costs and impaired health-related quality of life [6]. It is the leading cause of disability among chronic respiratory diseases and was the second leading contributor of Disability Adjusted Life Years (DALY) in 2016 [5]. In 2016, nearly 32% of global DALYs due to COPD occurred in India and COPD is responsible for 75.6% of total DALYs among chronic respiratory disease in India [5]. COPD-related mortality has been reported to be 39% from 2007-2017 [7]. Most of the existing data on COPD is from high-income countries, but 90% of deaths occur in low and middle-income countries [4]. Both India and China contributed to 33% of the world population and accounted for 66% of COPD mortality [5,8].

The number of COPD cases in India was a staggering 55.3 million in 2016 and is the second common cause of deaths due to NCD [5]. Evidence from India suggested the COPD prevalence increases with age and exponentially after 30 years of age. The estimated prevalence of COPD ranged from 0.1% to 0.9% between the age group of 5 years to 29 years while the incidence ranged from 1.6% to 28.3% in population above 30 years of age [5]. The prevalence of COPD varies across different regions and states of India. While the COPD prevalence in Bangalore was reported to be 4.36%, evidence from Delhi reported a prevalence of 10% [9,10] whereas the prevalence in Kerala was reported to be 6.19% among the general population [11]. Evidence from a multi-centric study further reported the prevalence of Chronic Bronchitis (CB) was 3.5% in population above 35 years [12]. A systematic review revealed the gender-wise variation in prevalence, where COPD rates in males ranged between 2% to 22% and that for females between 1.2 to 19% [13].

Risk factors for COPD include smoking, use of cooking fuel, biomass fuel and firewood, outdoor air pollution, increasing age, occupation, gender, pulmonary impairment after tuberculosis and socio-economic status among many others [14,15]. Older age, lower socio-economic status, level of education, poor knowledge about smoking consequences, and rural areas were found to be associated with smoking [16]. Pooled evidence from multiple studies showed the risk of COPD is higher with lower socio-economic status, air pollution, and exposure to environmental or occupation tobacco/dust [17-21].

In 2002, Global initiative for Chronic obstructive lung disease (GOLD) Science Committee was established with an aim to review published research on COPD management and prevention, and to evaluate the impact of research on recommendations on GOLD documents [22]. According to the GOLD criteria, spirometry is the gold standard diagnostic measure for confirming COPD with FEV1/FVC values of <70% [1]. However, the pulmonary function test (PFT) is not routinely performed during outpatient consultation leading to underestimating COPD cases especially during the early phases of the disease [23]. The data from low- and middle-income countries (LMIC) is underestimated due to the scarcity in diagnosing the disease using spirometric measurements, thus leading to under-reporting of COPD cases in LMICs [24]. A study conducted in Italy reported that only 56.2% of doctor-diagnosed COPD cases were confirmed via spirometry assessment. This is alarming as it endangers misdiagnosis of COPD, under-reporting of COPD which impedes early detection and treatment [25].

A national-level prevalence estimate for COPD in India is lacking. There is a dearth of evidence on the burden of COPD in the recent past in Indian population above 30 years along with the associated risk factors. The difference in the prevalence of COPD when diagnosed with spirometry compared to other measures is crucial for the development of guidelines. Studies reported difference between spirometry and non-spirometry methods for COPD prevalence. A higher prevalence of COPD was consistent with studies using spirometry method and under-estimation of COPD prevalence was observed in non-spirometric studies. This also suggests positive internal consistency with spirometry-based estimates [26]. The reasons for not employing spirometry could be attributed to lack of defined training cascade for diagnosis of COPD and limited availability of spirometry equipment in health facilities. Therefore, the objectives of this review are i) to estimate the prevalence of COPD among population above 30 years in India, ii) compare the prevalence of COPD among population diagnosed through spirometry and non-spirometry method and iii) to ascertain risk factors associated with COPD among the Indian population aged above 30 years.

## METHODOLOGY

A protocol was drafted prior to commencing the review and registered in the International Prospective Register of Systematic Reviews (PROSPERO) [27] which was later published [28].This systematic review is reported according to the Preferred Reporting Items for Systematic Reviews and Meta-Analyses (PRISMA) statement [29] and selection of studies is represented using revised PRISMA 2020 flow diagram [30].

### Search strategy and data sources

We first identified relevant medical subject headings (MeSH) from the MeSH browser [31] followed by identifying other key terms in consultation with the senior author (MPR) and a senior librarian from a Manipal Academy of Higher Education (MAHE). Search terms were combined using Boolean operators and a search strategy was constructed by NG, PN, AM and AV. Searches were carried out in Medline (PubMed), Cochrane, Scopus, Web of Science, CINAHL, and ProQuest databases. The following databases were identified as authors had access through their organization and these are comprehensive enough to culminate literature on COPD. The search strategy used in the PubMed database is presented in Table S1 in the **Online Supplementary Document**. The search was carried between 8thJuly, 2020 and 9th July 2020 by three authors (PN, NG and AM) and further verified by (AV and MPR). Reference lists of included articles were further scanned for potential inclusions. Search was restricted to publications in the English language and to articles published between the years 2000-2020 as evidence suggested that articles included from the past 20 years would not lead to loss of pertinent studies [32].

### Study selection

**Population:** Studies whose subjects were from India (permanent residents of India) aged 30 years and above. Studies whose target population was individuals with co-morbidities were excluded.

**Intervention and comparator:** None

**Outcome:** Prevalence of COPD, prevalence of COPD with spirometry as compared to non-spirometry methods and risk factors associated with COPD.

**Study designs**: Cross-sectional and cohort designs conducted among the Indian population aged above 30 years reporting a prevalence of COPD and(or) its risk factors were included.

### Screening

**Figure 1:**
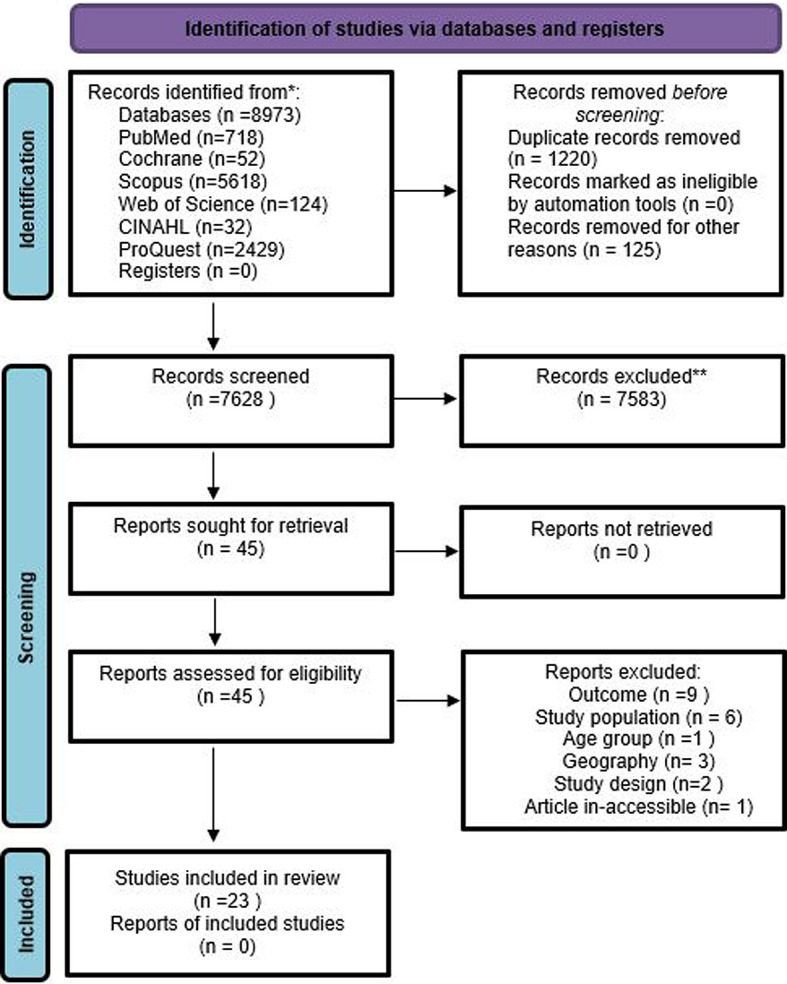
A detailed representation of the review process (PRISMA).

Articles were screened at two stages-i.e., Title-Abstract and full-text. On completing the search in different databases, records were imported into Rayyan software [33]. Two authors (PN, AM) independently screened articles for potential inclusion. In-case of conflict, a consensus was reached in consultation with the third authors (AV, NG). Reasons for exclusion were recorded and a list of excluded articles is provided in Table S2 of the **Online Supplementary Document**. A PRISMA chart shows the volume of articles at different stages of this review, **Figure 1**.

### Data extraction

Data were extracted by two authors independently (NG, AV) using a pre-tested data extraction form in Microsoft Excel, 2007. The form was pilot tested on five percent of the studies (8 studies) and once modified for inclusion of additional fields, data extraction was completed. The data extraction form was reconciled by AM, PN and finalized. Major domains under which the data was extracted were: (i) study characteristics, (ii) methodological characteristics, (iii) study definitions, (iv) factors identified (eg, disease risk factors like smoking status, previous exposure of smoking, type of data, a measure of association calculated), and (v) other important information such as the definition of exposure and outcome, mechanisms for the ascertainment of exposure and outcome, and controlled confounders. If authors did not clearly define the study setting (urban, rural, or occupational) in the included studies then it was considered as a mixed setting. The prevalence of COPD including chronic bronchitis and emphysema, was independently evaluated from spirometric and non-spirometric sets of studies as described by Adeloye et al [26].

### Quality assessment and reporting quality

The quality of included studies was assessed independently by two authors (AV, NG), and a consensus was reached between the two authors in case of disagreements. For the included studies, we used the modified New Castle Ottawa Scale (NCOS) for assessing their quality [34,35]. Reporting quality of a study often plays a vital role in influencing decision maker’s opinion as lack of detailing results in not utilizing the evidence [36]. Similarly, inadequate reporting may also be a challenge for the synthesis of evidence [37], we also examined the reporting quality of these included observational studies according to “The Strengthening the Reporting of Observational Studies in Epidemiology (STROBE)” statement [38]. We have included the quality of reporting and study quality in Table S3 and Table S4 of the **Online Supplementary Document** to facilitate clarity of the inferences we have drawn from the study.

### Meta-analysis

Meta-analysis was performed to estimate the prevalence of COPD diagnosed through spirometry and non-spirometry methods using STATA 13.1 [39]. Data were graphically represented using forest plots. Publication bias was considered statistically significant when a *P* value of less than 0.10 was obtained. Heterogeneity across studies was assessed using the χ2 test for heterogeneity with a 5% level of statistical significance [40] and using the *I^2^* statistic, for which values greater than 50% implied moderate heterogeneity [41].

### Deviations from the protocol

We had proposed to use the NCOS for assessing the quality of non-randomized studies, but we later used the modified NCOS. We had also proposed to ascertain risk factors associated with COPD, while we could not achieve this objective as relevant details were not expressed in the form of odds ratios and relative risks in the majority of studies to aid further quantification.

## RESULTS

The initial search identified 8973 records of our interest. Based on our inclusion criteria, 45 records were included at the title and abstract screening stage. Of the 45 included studies (following Ti-Ab), 22 records were excluded and the list of excluded studies along with reason is provided in Table S2 of the **Online Supplementary Document**, and 23 studies were included for data extraction (**Table 1**). No other study was identified through hand search of relevant bibliographies.

**Table 1:** List of included studies

Study ID	Population	Study setting	Method of diagnosis	Outcome	Study design
Parasuramalu 2014 [9]	Aged more than 35 y	Rural area of Karnataka	Spirometry	COPD prevalence – 4%	Cross-sectional
Sinha 2017 [10]	Aged 46 y and above	Urban area of Delhi	Spirometry	COPD prevalence – 10.10%	Cross-sectional
Viswanathan 2018 [11]	Aged 35 y and above	Kerala	Non- Spirometry	CB prevalence – 6%	Cross-sectional
Jindal 2012 [12]	Aged 30 to >65 y	Rural and Urban area of Rajasthan, Gujarat, Maharashtra, Karnataka, Kerala, TN, Andhra Pradesh, Odisha, WB, Assam, HP	Non- Spirometry	CB prevalence – 3.49%	Cross-sectional
Sabde 2008 [17]	Aged 20- 54 y	Urban area of Maharashtra	Non- Spirometry	CB prevalence - 5.90%	Cross-sectional
Rana 2018 [18]	Mean age is 38 y	West Bengal	Spirometry	COPD prevalence- 24.60%	Cross-sectional
Sharma 2019 [19]	Not Available	Punjab	Spirometry	COPD prevalence- 3.17%	Cross-sectional
Mahmood 2017 [20]	Mean age is more than 50 y	Uttar Pradesh	Spirometry	COPD prevalence- 7%	Cross-sectional
Panigrahi 2018 [21]	Aged 35-49 y	Rural area of Maharashtra	Spirometry	COPD prevalence- 7.3%	Cross-sectional
Mahesh 2009 [42]	Aged 40 y and above	Rural area of Karnataka	Spirometry	COPD prevalence- 7.1%	Cross-sectional
Banjare 2014 [43]	Aged 60 y and above	Rural area of Odisha	Non- Spirometry	COPD prevalence- 20%	Cross-sectional
					
Mahesh 2018 [44]	Aged 35-80 y	Rural area of Karnataka	Spirometry	CB prevalence- 7.7%	Cohort
Kashyap 2020 [45]	Aged 35 y and above	Uttar Pradesh	Non- Spirometry	CB prevalence- 13%	Cross-sectional
Koul 2016 [46]	Aged 40 y and above	Rural area of Jammu & Kashmir	Spirometry	COPD prevalence- 8.40%	Cross-sectional
Mahesh 2013 [47]	Aged 30 y and above	Rural area of Karnataka	Non- Spirometry	CB prevalence- 3.40%	Cross-sectional
					
Rajavel 2020 [48]	Mean age is 39 y	Rural area of Rajasthan	Spirometry	Emphysema prevalence- 3.70%	Cross-sectional
Chopra 2017 [49]	Aged 31 y and above	NA	Spirometry	COPD prevalence- 9%	Cross-sectional
Praveen 2018 [50]	Aged more than 30 y	Urban area of Hyderabad	Spirometry	COPD prevalence- 11%	Cross-sectional
Christopher 2020 [51]	Aged more than 30 y	Rural area of Tamil Nadu	Spirometry	COPD prevalence- 9%	Cross-sectional
Mahesh 2014 [52]	Aged more than 30 y	Rural area of Karnataka	Non- Spirometry	CB prevalence- 11%	Cross-sectional
Jindal 2006 [53]	Aged more than 35 y	Rural and Urban area of Chandigarh, Delhi, Kanpur and Bangalore	Non- Spirometry	COPD prevalence- 4.10%	Cross-sectional
Medhi 2006 [54]	Aged more than 60 y	Urban area of Assam	Non- Spirometry	COPD prevalence- 7.60%	Cross-sectional
Johnson 2011 [55]	Aged more than 30 y	Rural area of Tamil Nadu	Spirometry	COPD prevalence- 2.44%	Cross-sectional

y – years

Of the 23 studies, 22 followed cross-sectional study design [9-12,17-21,42,43,45-55] while one study had employed cohort study design [44]. Overall, the sample size from the included studies was 80 138 and represented 19 states and union territories of India, out of the 36 states and union territories [56]. Included studies nearly represented the wide geographic coverage of India as studies were conducted in Southern states – Karnataka [9,12,42,44,47,52,53], Tamil Nadu [12,51,55], Kerala [11,12], Telangana[19] and Andhra Pradesh [50]; Northern states – Uttar Pradesh [45,46,53], Delhi [10,53], Punjab [19], Himachal Pradesh [12], Chandigarh [53] and Jammu and Kashmir [46]; Western states – Maharashtra [12,17,21] Gujarat [12]; and Eastern states – Odisha [12,43], Assam [12,54] and West Bengal [12,18]. The states were geographically classified based on the Government of India’s official notification [57]. Two multicentric studies were conducted in 11 states [53] and 4 states [12] respectively.

Of the 23 included studies, nearly 50% [12] studies were conducted in rural setting [9,11,21,42-44,46-48,51,52,55], 5 in urban setting [10,17,18,50,54], 2 studies were conducted in mixed setting [12,53],4 studies did not reveal their study region [19,20,45,49] and are further considered as studies conducted in mixed region. Nearly 60% studies (14) used spirometry as the diagnostic tool for COPD [9,10,18-21,42,44,46,48-51] whereas 40% (9) studies used non spirometric method for COPD diagnosis [11,12,17,43,45,47,52-54]. Overall, 15 studies reported COPD as their outcome [9,10,18-21,42,43,46,49-51,53-55], other 7 studies reported chronic bronchitis [11,17,44,45,47,52,53] and one study reported emphysema as their outcome [48].

Although studies reported a data on population below 30 years, we extracted data of the sample aged above 30 years. Majority of the studies included both genders, while only 3 studies included only males [45,50,52] and only females [21,47,55]. Nine studies were conducted in 2017-18 [14,18,19,28-31,33,34] and two studies were conducted recently in 2020 [48,51].

### Pooled prevalence estimates for COPD

**Figure 2:**
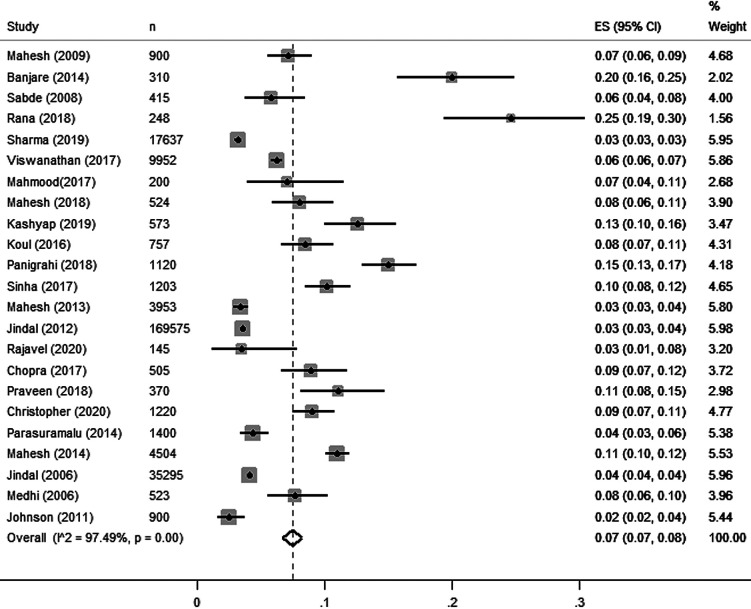
Forest plot of the included studies.

Pooled prevalence estimated for all studies is presented in **Figure 2**. Overall, 23 estimates for COPD [studies including that reported either COPD – including both chronic bronchitis, or emphysema) with the sample size of 80 138 yielded a pooled estimate of 7% or 0.07 (95% confidence interval (CI) = 0.07, 0.08). There was higher heterogeneity (97.8%) observed among included studies as they followed observational study design.

### Prevalence of COPD diagnoses with spirometry method

Pooled estimates of COPD diagnosed through spirometry reported a prevalence of 8% or 0.08 (95% CI = 0.06, 0.09) from 14 studies [9,10,18-21,42,44,46,48-50,55,58] which includes 27 341 study participants across 11 states and union territories. Prevalence of COPD was reported in 12 studies [9,10,18-21,42,46,49,50,55,58] and one study reported the prevalence of chronic bronchitis [44] and emphysema [48] respectively.

### Prevalence of COPD diagnoses with the non-spirometry method

Non-spirometry method includes diagnosis through pretested structured questionnaire and validated tools [11,12,17,43,45,47,52-54]. In this review, pooled estimates of COPD diagnosed through non spirometry method reported the overall prevalence of 7% or 0.07 (95% CI = 0.06, 0.09) from 9 studies [11,12,17,43,45,47,52-54] which includes 52 797 study participants across 14 states and union territories. Among these 9 studies, only 3 studies reported COPD [43,53,54] and 6 studies reported chronic bronchitis as their outcome [11,12,17,45,47,52].

### Exposure to risk factors

Majority of study participants had been exposed to risk factors like smoke and smokeless tobacco in which prevalence of any form of tobacco use ranges from 5.6% to 100%, only 3 studies refute the exposure of smoke among study participants [47,48,55] and one study [49] did not mention the smoking status of study participants. Different exposure to smoke includes bidi, cigarette smoke, passive smoke at home or workplace. Half of the studies have study participants exposed to biomass fuel once in their lifetime due to the cooking inside the house using firewood, cooking fuel combustion or kerosene [10-12,20,21,42,44,46,47,55]. Consumption of alcohol, occupational exposure in rice mill and dust, history of TB, air pollution, and age were considered as the risk factor for COPD [12,19,21,42-44,46,47,50,53]. Only 8 studies reported the socio-economic status among study participants [10,18,20,21,44,48,53,58] and only single study reported lower socio-economic status as the risk factor for COPD [20].

### Case definition

The definition of COPD was a complicated task in estimating the prevalence of COPD. As described in the Global Initiative for Chronic Obstructive Lung Disease (GOLD) criteria handbook, “COPD is a common, preventable, and treatable disease that is characterized by persistent respiratory symptoms and airflow limitation that is due to airway and/or alveolar abnormalities usually caused by significant exposure to noxious particles or gases. The chronic airflow limitation is caused by a mixture of small airways disease (e.g. chronic bronchitis) and parenchymal destruction (emphysema)”[1]. Chronic bronchitis is “defined as cough with expectoration occurring on most days for at least 3 months of the year for at least 2 consecutive years” [59]. In this review, only 11 studies referred the GOLD definition for the diagnosis of COPD [9-11,19,20,42,44,49,50,55,58]. The definition used in two studies was based on cough and sputum [21,45] and only one study [9] used the Medical Research Council, UK definition [60]. Seven studies reported the prevalence of chronic bronchitis and not COPD and used the case definition of chronic bronchitis [11,12,17,44,45,47,52].

### Risk factors for COPD

In majority of studies, the most common significantly associated risk factors in India were found to be active and passive smoking and biomass fuel exposure [9-12,20,21,42-47,49,50,52-54,58,61,62]. Both first-hand/active smoking and passive/shand smoke at office and home in the form of cigarette/bidis, cigars were prevalent and were associated with COPD among study participants. Smokeless tobacco includes betel quid with tobacco, khaini or tobacco, gutka or tobacco lime, cheroots and pan masala were other associated risk factors for COPD. These studies also referred to the age of starting consumption of tobacco, years of smoking, frequency, current smoking status, pack-years and smoking index as risk factors for COPD. In a single study, alcohol consumption was also found to be higher among the participants aged 35 years and above but there is a negligible association between alcohol and chronic bronchitis [45]. Biomass fuel exposure in terms of use of wood in the house, solid fuel, gas, kerosene or cooking fuel, coal was considered as the second most associated risk factor for COPD including both chronic bronchitis and emphysema in the majority of included studies [10-12,21,42,46,47,50,52,54,58,61].

Other risk factors which are very commonly found in India are occupational and environmental exposure to dust [10,11,17,18,45,47,53-55]. It was seen that the prevalence of CB was significantly higher among street sweeper which is due to occupational exposure to dust [17]. One more study in this review reported the prevalence of COPD was significantly higher among people who are having experience of working in rice mills for over 9 hours and 20 years [18].

Individuals with COPD are vulnerable to the detrimental effects of indoor and outdoor air pollution. Indoor air pollution in the form of combustion of solid fuels is a major source of air pollutants in developing countries. A study conducted in Ludhiana (Punjab), one of the most polluted industrial town in India reported the high prevalence of COPD in the hotspots (71% more cases in industrial area) and suggested the potential association with industrial pollutants with COPD [19].

Interestingly, few studies reported long-standing previously diagnosed Asthma and family history of respiratory disease as the associated risk factors of COPD among study participants [12,20,46,58]. Low socio-economic status, pulmonary tuberculosis, educational status and increasing age were also evident and associated with the occurrence of COPD among individuals in India [10-12,21,46-49,53].

### Quality appraisal for included studies

The quality of included studies was evaluated by two authors independently (AV and NG) according to Newcastle-Ottawa scales (NCOS) adapted to cross-sectional and cohort studies (Appendix S1 in the **Online Supplementary Document**). The following criteria were considered while grading the quality: a score of 9 or above as very good quality, a score between 7-9 as good quality, score between 5-6 as satisfactory quality, and a score below 5 considered as unsatisfactory. Based on the above said criteria, only one study was scored as a very good quality [46]. A total of five studies were considered as good quality studies [9,12,20,41,50]. In this review, 10 studies fall under this satisfactory quality [10,11,43,45,48,50,52,55,58,63] and seven studies were unsatisfactory based on established criteria [17-20,42,49,54].

## DISCUSSION

This systematic review reports findings from the published studies that focused on COPD prevalence and its risk factors conducted among population above 30 years in India between 2000 and 2020. Using the meta-analysis process, the current study revealed a COPD prevalence to be 7.0% using the estimates from 23 studies where the cumulative study participants were 80 138 in India. Our results indicate a lower prevalence of COPD than the global prevalence ranging from 10.7% to 12.1% [24]. The pooled prevalence of COPD reported in the current study is lower than reported (8.80%) in the South-East Asia region but is similar to the spirometry diagnosed COPD prevalence [24]. In contrast, we found a high prevalence of COPD than that reported from a meta-analysis (5.87%) of the studies from China [64] and lower than that reported in Bangladesh (12.5%) [65]. Notably, the stratified prevalence estimation showed a higher prevalence of COPD (8%) among those diagnosed with spirometry as compared to the diagnosis made using non-spirometry method (7%). These findings are similar to that of meta-analysis estimates from Africa that showed higher COPD prevalence on spirometry (13.4%) data compared to non-spirometry one (4.0%) [26]. Moreover, in comparison to our findings, studies from Nepal – 15.4% [66], Pakistan – 11.31% [67], Sri Lanka – 16.4% [68] and Bangladesh – 13.5% [69] reported higher prevalence. On comparing the prevalence of other studies with this review, we observed variation with the prevalence of COPD in other study settings, including South Asian countries could be because of differences in geographical settings, criteria for COPD diagnosis, exposure to COPD risk factors, diversity of COPD diagnostic definitions, sampling method and study population. Evidence shows that 50%-90% of people with COPD remained undiagnosed due to lack of availability or accessibility of a spirometer or the trained health professionals to diagnose the COPD [70,71]. In India, programs to combat NCDs are primarily focused on diabetes, cardiovascular disease, and hypertension, however, due importance is warranted to diagnose and manage COPD [71]. In addition, the National Programme for Prevention and Control of Cancer, Diabetes, Cardiovascular Diseases and Stroke (NPCDCS) launched by the government of India screen patients for COPD at the district hospital only but not on peripheral level viz. sub centre and primary health centre level, reflecting under-reporting of the COPD cases [72]. Therefore, policy makers need to prioritize COPD screening under national health program for NCDs and the findings of this study warrant the need for a national survey to estimate the actual burden of COPD in India.

In the present study, we could not provide stratified COPD prevalence for age, smoking, gender, and stages of COPD because the included studies did not provide information on these variables. This brings attention to the lack of availability of good recording of COPD-related data in developing countries including India [24,73]. This suggests the need for well-designed epidemiological studies that can capture both socio-demographic, exposure, and clinical variables in order to draw persuasive conclusions that can guide COPD screening and management in India. It was also seen in this review that the robust evidence on COPD estimates based on standard definition as defined by GOLD is lacking. There was a considerable variation in the diagnostic criteria of COPD and study methodology techniques across different studies. Majority of studies were conducted in a community setting whereas limited evidence is available to demonstrate the relationship between occupational exposure and chronic respiratory diseases. [74].

In the current review, both active and passive smoking, environmental tobacco smoke (ETS) exposure are found to be associated with COPD but separate estimates among smokers were not mentioned in the majority of the studies which runs parallel to the findings of the review conducted by Halbert [75] and Jindal in 2006 [76]. This could be due to the methodology rigor and findings of included studies were not discussed in detail and sociodemographic data of the study participants could be lacking. Apart from the health effects of smoking, COPD has a negative impact on an individual’s health expenditure. An average COPD patient in India spent nearly 15% of his salary on consuming smoking products and 30% on disease management [76]. We observed TB as one of the risk factors for COPD. Evidence from previous studies reveal higher prevalence of COPD in individuals with TB that is indicative of association between COPD and TB [15,49].

In this review, biomass fuel exposure is another significantly associated risk factor for COPD further supported by Pathak’s review in 2019 which highlighted the significant impact of indoor air pollution in terms of biomass fuel exposure on COPD in LMICs [77]. Cooking practices that rely on biomass fuel in poorly ventilated kitchen may leads to high exposure to particulate matter which is likely to contribute to the COPD burden. An estimated 925 000 premature deaths take place in India annually from indoor air pollution attributed to cooking [78]. Inadequate ventilation and domestic exposure to smoke were underscored in another meta-analysis from India [79]. With an increase in the use of clean fuel, a reduction in mortality from COPD has been noted [80]. This highlights the need for an improved cooking stoves (chulhas) or shifting to smokeless cooking (like LPG) that provide the complete combustion of biomass fuel, hence doesn’t cause indoor air pollution. Air pollution seems to be a greater contributor than smoking to COPD burden in India [5]. Government of India also defined the air quality in terms PM 2.5 values in terms of good (0-30 μg/m^3^), satisfactory (30-60 μg/m^3^), moderate (60-90 μg/m^3^), poor (90-120 μg/m^3^), very poor (120-150 μg/m^3^) and severe (250-380 μg/m^3^) [81]. Our review showed occupational and environmental exposure (dusty outdoor environment, working in rice mills and street) attributed to the prevalence of COPD. Evidence suggests that air pollution is the largest risk factor for lung conditions in developing countries like India where exposures to PM 2.5 – fine particulate matter is worrying [82,83]. Additionally, research from India showed that exposure to Rice husk dust is associated with a decrease in lung function and an increase in respiratory symptoms and long-term exposure may lead to the development of COPD [84,85].

This suggests the need to effectively implement multisectoral policies to combat outdoor air pollution and workplace air pollution. There are policies such as National Clean Air Programme (NCAP), policies to push CNG and PNG in cities, reducing vehicular pollution, smoking in public places and stubble burning strategies like urbanization, transportation, industrialization, power generation, and agricultural activities to reduce the levels of both indoor and outdoor pollution, however the implementation of such policies are questionable [86-89]. Our findings also suggest the need for COPD sensitive health literacy program focused on early screening and primary prevention of risk factors for COPD, which may help early initiation of self-management practices, that are crucial for better quality of life [90-93].

### Strengths and limitations of this review

The study provides pooled prevalence of COPD in general, and the prevalence of COPD diagnosed with spirometry and non-spirometry method. The study further validates the risk factors for COPD and highlights those indigenous to the region such as occupational (street sweeping and working in rice mills) and smokeless tobacco products such as betel quid with tobacco, khaini or tobacco, gutka (a sweetened mixture of chewing tobacco, betel nut, and palm nut, originating in India as a breath freshener) or tobacco lime, cheroots and pan masala which are commercially marketed and available in India. We included studies published in the English language and could have missed few potential articles that were published in other languages. One should be cautious that we have excluded COPD with comorbidity in this study which may have underestimated the true prevalence of COPD among population above 30 years in India.

## CONCLUSION

This study is the first to report a pooled prevalence of COPD considering both spirometry and non-spirometry measurement among population above 30 years in India. In conclusion, there is a need for multi-centric studies considering both rural and urban population, equipped with standard definition and diagnostic procedure for COPD. Also, there is a need to identify risk factors relevant to India, which this review tried to capture but very few studies expressed these as measures of association and quality of these studies was low.

## Additional material


Online Supplementary Document
