# Supplementary material for: Prevalence of COPD among population above 30 years in India: A systematic review and meta-analysis
Source: J Glob Health. 2021 Aug 21;11:04038. doi: 10.7189/jogh.11.04038 (PMC8397327; doi:10.7189/jogh.11.04038)
Supplement: Online Supplementary Document [file jogh-11-04038-s001.pdf]

Table S1: SEARCH Strategy for PubMed database

Sl no	Search Strategy
**6**	#1 AND #3 AND #2/OR #2 AND #4
**5**	#1 AND #3 AND #2
**4**	((((((((risk factor) OR (determinant*)) OR (risk*)) OR (predictor*)) OR (relative risk)) OR (odds ratio)) OR (attributable risk)) OR (population attributable fraction)) OR (factor*)
**3**	((((((((((((((Chronic obstructive disease) OR (Chronic obstructive pulmonary disease)) OR (chronic obstructive airway disease)) OR (chronic obstructive lung disease)) OR (COPD)) OR (COAD)) OR (bronchitis)) OR (chronic bronchitis)) OR (exacerbation emphysema)) OR (airway obstruction)) OR (Lung diseases, Obstructive)) OR (Emphysema)) OR (Pulmonary disease, Chronic obstructive)) OR (COBD)) OR (AECB)
**2**	((((((((((((((((((((((((((((((((((((((India) OR (Andhra Pradesh)) OR (Arunachal Pradesh)) OR (Assam)) OR (Bihar)) OR (Chhattisgarh)) OR (Goa)) OR (Gujarat)) OR (Haryana)) OR (Himachal Pradesh)) OR (Jharkhand)) OR (Karnataka)) OR (Kerala)) OR (Madhya Pradesh)) OR (Maharashtra)) OR (Manipur)) OR (Meghalaya)) OR (Mizoram)) OR (Nagaland)) OR (Odisha)) OR (Orissa)) OR (Punjab)) OR (Rajasthan)) OR (Sikkim)) OR (Tamil Nadu)) OR (Telangana)) OR (Tripura)) OR (Uttar Pradesh)) OR (Uttarakhand)) OR (West Bengal)) OR (Andaman and Nicobar)) OR (Chandigarh)) OR (Daman and Diu)) OR (Dadar and Nagar Haveli)) OR (Delhi)) OR (Jammu and Kashmir)) OR (Ladakh)) OR (Lakshadweep)) OR (Puducherry)
**1**	(closed cohort studies) OR (cohort analysis) OR (cohort stud*) OR (historical cohort studies) OR (concurrent studies) OR (incidence studies) OR (cross sectional studies) OR (cross sectional survey) OR (disease frequency surveys) OR (prevalence studies)

Table S2: Reason for studies excluded at full text stage

Study ID	Title	Reason for exclusion
Apte 2016	Prevalence of cough and its associated diagnosis among 204,912 patients seen in primary care (PC) in India	Based on outcome
Bajpai 2019	Clinical, demographic and radiological profile of smoker COPD versus non-smoker COPD patients at a tertiary care center in North India	Based on study population
Singh A 2017	Indoor air pollution and its association with poor lung function, microalbuminuria and variations in blood pressure among kitchen workers in India: a cross-sectional study	Based on outcome
Senthilnathan 2016	Early detection of COPD in asymptomatic smokers using spirometry	Based on the age group
Patil 2012	Prevalence of obstructive airway disease by spirometric indices in non-smoker subjects with IHD and HTN	Based on study population
Wang 2018	Practice Patterns for Chronic Respiratory Diseases in the Asia-Pacific Region: A Cross-Sectional Observational Study	Based on study area
Sahab 2013	A cross sectional study of Pulmonary function test in street cleaners in Aligarh, India	Based on outcome
Mazumder 2007	Arsenic and non-malignant lung disease	Based on outcome
Khafaie 2017	Air pollution and respiratory health among diabetic and non-diabetic subjects in Pune, India—results from the Wellcome Trust Genetic Study	Based on study population
Chandra 2018	Tuberculosis and other chronic morbidity profile of sewage workers of Delhi	Based on study population
Deo 2009	Periodontitis as a potential risk factor for chronic obstructive pulmonary disease: A retrospective study	Based on outcome
Chhabra 2001	Ambient Air Pollution and Chronic Respiratory Morbidity in Delhi	Based on study population
Stephen2018	High Prevalence of Chronic Respiratory Symptoms among Autorickshaw Drivers of Urban Puducherry, South India	Based on outcome
Ghoshal 2016	The burden of segregated respiratory diseases in India and the quality of care in these patients: Results from the Asia-Pacific Burden of Respiratory Diseases study	Based on study population
Cho 2016	Respiratory disease in the Asia-Pacific region: Cough as a key symptom	Based on study area
Mazumder 2009	Chronic arsenic toxicity: Studies in West Bengal, India	Based on outcome
Hystad 2019	Health Effects of Household Solid Fuel Use : Findings from 11 Countries within the Prospective Urban and Rural Epidemiology Study	Based on study area
Sehgal 2014	Disease burden due to biomass cooking-fuel related household air pollution among women in India	Based on study design
Bose 2013	CD14 C-159T polymorphism and its association with chronic lung diseases: A pilot study on isocyanate exposed population of Central India	Based on outcome
Sur 2007	A study on smoking habits among slum dwellers and the impact on health and economics	Article inaccessible
Kumar 2004	Association of Outdoor Air Pollution with Chronic Respiratory	Based on outcome

	Morbidity in an Industrial Town in Northern India	
Chakraborty 2009	Chronic Exposures to Cholinesterase Pesticides Adversely Affect Respiratory Health of Agricultural Workers in India	Based on study design

Table S3: Risk of Bias Assessment for cross sectional studies- Modified New Castle Ottawa scale

Study ID	Selection				Comparability based on design and analysis	Outcome		
	Representative ness of the sample	Sample size	Non-respondents	Ascertainment of the exposure (risk factor)		Assessment of the outcome	Statistical test	Total
Mahesh 2009				**				2
Banjare 2014	*	*		*		*	*	5
Sabde 2008							*	1
Rana 2018	*	*					*	3
Sharma 2019				*				1
Viswanathan 2017	*	*		**		*	*	6
Mahmood 2017			*		*			2
Stephen 2018	*	*		**		*		5
Kashyap 2019	*	*		*		*	*	5
Koul 2016	*	*	*	**	**	*	*	9
Panigrahi 2018	*	*		**	*	*	*	7
Sinha 2017	*	*		**		*		5
Mahesh 2013	*	*		**	*	*	*	7
Jindal 2012	*	*	*	**	*	*	*	8
Rajavel 2020	*	*	*	*		*	*	6
Chopra 2017	*	*		**				4
Praveen 2018	*	*		**			*	5
Christopher 2020	*	*		**		*	*	6
Parasuramalu 2014	*	*		**	**	*	*	8
Mahesh 2014	*	*		**	*	*	*	7
Jindal 2006	*	*		**		*	*	6
Medhi 2006						*	*	2
Johnson 2011	*	*			**		*	5

Table S4: Risk of Bias Assessment for a cohort study- Modified New Castle Ottawa scale

Study ID	Selection				Comparability	Outcome		
	Representativeness of exposed cohort	Selection of nonexposed cohort	Ascertainment of exposure	Outcome of interest was not present at start of study	Adjustment and assessment of outcome	Follow-up length	Loss to follow-up rate	Total
Mahesh 2018	*	NA	*	*	*	4 years	31.5 %	4

Appendix S1

Newcastle-Ottawa Scale adapted for cross-sectional and cohort studies

Note: A study can be awarded a maximum of one star for each numbered item within the Selection and Exposure categories. A maximum of two stars can be given for Comparability.

Selection:

1. Representativeness of the sample:

a. Truly representative of the average in the target population. * (all subjects or random sampling)

b. Somewhat representative of the average in the target group. * (non-random sampling)

c. Selected group of users/convenience sample.

d. No description of the derivation of the included subjects.

2. Sample size:

a. Justified and satisfactory (including sample size calculation). *

b. Not justified.

c. No information provided

3. Non-respondents:

a. Proportion of target sample recruited attains pre-specified target or basic summary of non-respondent characteristics in sampling frame recorded. *

b. Unsatisfactory recruitment rate, no summary data on non-respondents.

c. No information provided

4. Ascertainment of the exposure (risk factor):

a. Vaccine records/vaccine registry/clinic registers/hospital records only. **

b. Parental or personal recall and vaccine/hospital records. *

c. Parental/personal recall only.

Comparability: (Maximum 2 stars)

1. Comparability of subjects in different outcome groups on the basis of design or analysis. Confounding factors controlled.

a. Data/ results adjusted for relevant predictors/risk factors/confounders e.g. age, sex, time since vaccination, etc. **

b. Data/results not adjusted for all relevant confounders/risk factors/information not provided.

Outcome:

1. Assessment of outcome:

a. Independent blind assessment using objective validated laboratory methods. **

b. Unblinded assessment using objective validated laboratory methods. **

c. Used non-standard or non-validated laboratory methods with gold standard. *

d. No description/non-standard laboratory methods used.

2. Statistical test:

a. Statistical test used to analyse the data clearly described, appropriate and measures of association presented including confidence intervals and probability level (p value). *

b. Statistical test not appropriate, not described or incomplete.

MODIFIED NEWCASTLE - OTTAWA QUALITY ASSESSMENT SCALE FOR COHORT STUDIES
Note: A study can be awarded a maximum of one star for each numbered item within the Selection and Outcome categories. A maximum of two stars can be given for Comparability

SELECTION
1 Representativeness of the exposed cohort
a) Consecutive eligible participants were selected, participants were randomly selected, or all participants were invited to participate from the source population*
b) Not satisfying requirements in part (a), or not stated.

2 Selection of the non-exposed cohort
a) Selected from the same source population*
b) Selected from a different source population
c) No description

3 Ascertainment of exposure
a) Structured injury data (e.g. record completed by medical staff)*
b) Structured interview*
c) Written self-report
d) No description

4 Demonstration that outcome of interest was not present at the start of the study
a) Yes*
b) No or not explicitly stated

COMPARABILITY
1 Comparability of cohorts on the basis of the design or analysis
a) Study controls for previous injury*
b) Study controls for age*
Note: Exposed and non-exposed individuals must be matched in the design and/or confounders must be adjusted for in the analysis. Alone statements of no differences between groups or that differences were not statistically significant are not sufficient.

OUTCOME
1 Assessment of outcome
a) Independent or blind assessment stated, or confirmation of the outcome by reference to secure records (e.g. imaging, structured injury data, etc.)*
b) record linkage (e.g. identified through ICD codes on database records)*
c) Self-report with no reference to original structured injury data or imaging
d) No description

2 Was follow-up long enough for outcomes to occur?
a) Yes (≥3 months)*
b) No (<3 months)

3 Adequacy of follow up of cohorts
a) Complete follow up – all participants accounted for*

b) Subjects lost to follow up unlikely to introduce bias (<15% lost to follow up, or description provided of those lost*)
c) Follow up rate <85% and no description of those lost provided
d) No statement

SCORE:
Very Good Studies: 9-10 points
Good Studies: 7-8 points
Satisfactory Studies: 5-6 points
Unsatisfactory Studies: 0 to 4 points
This scale has been adapted from the Newcastle-Ottawa Quality Assessment Scale for cohort studies to provide quality assessment of cross sectional studies[1].

[1]Herzog R, et al. Is Healthcare Workers' Intention to Vaccinate Related to their Knowledge, Beliefs and Attitudes? A Systematic Review. *BMC Public Health* 2013 **13**:154
